# Cutaneous Manifestations in COVID-19: Report on 31 Cases from Five Countries

**DOI:** 10.3390/biology10010054

**Published:** 2021-01-13

**Authors:** Carmen Rodriguez-Cerdeira, Brianda I. Uribe-Camacho, Lianet Silverio-Carrasco, Wennia Méndez, Ashwini R. Mahesh, Anakaren Tejada, Angelica Beirana, Erick Martinez-Herrera, Alfonso Alba, Roberto Arenas, Jacek C. Szepietowski

**Affiliations:** 1 Efficiency, Quality, and Costs in Health Services Research Group (EFISALUD), Galicia Sur Health Research Institute (IIS Galicia Sur), SERGAS-UVIGO, 36213 Vigo, Spain; carmencerdeira33@gmail.com (C.R.-C.); brivette20@gmail.com (B.I.U.-C.); lsilverio25@gmail.com (L.S.-C.); erickMartinez_69@hotmail.com (E.M.-H.); alfonsoalba@e-icm.net (A.A.); rarenas98@gmail.com (R.A.); 2 Dermatology Department, Hospital Vithas Ntra. Sra. de Fátima, 36206 Vigo, Spain; 3 University Campus, University of Vigo, 36310 Vigo, Spain; 4 Department of Internal Medicine, General Hospital, Atizapan 52975, Mexico; 5 Dermatological Institute and Skin Surgery “Dr. Huberto Bogaert Díaz”, Santo Domingo 1090, Dominican Republic; wennia_mendez@hotmail.com (W.M.); anakaren_t21@hotmail.com (A.T.); 6 Institute of Cutaneous Medicine, C1425 ASU Buenos Aires, Argentina; 7 Polyclinic National Centre, Santo Domingo 11517, Dominican Republic; 8 Oncoderm Clinic, Bangalore 560053, India; therightdiagnosis@gmail.com; 9 Dermatological Center Dr. Ladislao de la Pascua (CDP), Mexico City 06780, Mexico; angelicabeirana@hotmail.com; 10 Research Unit, Hospital Regional de Alta Especialidad de Ixtapaluca, Mexico City 56530, Mexico; 11 Institute of Cellular and Molecular Studies—ICM, 27003 Lugo, Spain; 12 Dermatology Department (Section Mycology) & Research Unit, General Hospital “Dr. Manuel Gea González”, Mexico City 14080, Mexico; 13 Department of Dermatology, Venereology and Allergology, Wroclaw Medical University, 1 Chalubinskiego Street, 50-368 Wroclaw, Poland

**Keywords:** COVID-19, skin lesions, maculopapular rash

## Abstract

**Simple Summary:**

This large international registry-based case series contributes to the emerging evidence that skin lesions are one of the important clinical manifestations of COVID-19. Distal ischemic and necrotic lesions, and livedo racemosa usually appears in severe cases and in final disease stages. Pseudochilblains are generally associated with a more benign clinical course. Cutaneous manifestations associated with COVID-19 probably reflect the activation of pathogenic pathways by the virus or a response to inflammatory processes, vascular or systemic complications, or even treatments. Clinical doctors, in general, must be familiar with the cutaneous manifestations of COVID-19 since they may be the only manifestation of the disease in some cases. Early recognition of the cutaneous manifestations of COVID-19 can enable early diagnosis, or guide prognosis and treatment.

**Abstract:**

The increasingly frequent cutaneous manifestations of coronavirus disease (COVID-19) remain to pose a problem to clinicians. Herein, we aimed to describe the clinical and pathological findings of skin lesions in patients with COVID-19. The case series, which was based on the International Dermatological Registry circulated to dermatologists worldwide, was conducted across organizations and societies belonging to five different countries. We documented 31 patients with dermatologic manifestations associated with COVID-19, including maculopapular rashes (16.10%), urticarial lesions (26.80%), pseudochilblains (22.60%), petechiae/purpura (6.50%), distal ischaemia and necrosis (6.50%), livedo racemosa (12.90%), and others (9.70%). Twenty-six cases (83.90%) were qRT-PCR-confirmed COVID-19 cases, two (6.50%) were serologically confirmed, while two others (9.7%) were suspected cases owing to previous contact with COVID-19-positive patients. Therefore, our findings indicate that a febrile rash or even a rash in an afebrile state in the early stages of the disease may be the only clinical manifestation of COVID-19. In the future, we recommend close monitoring of all patients with skin lesions not attributable to other causal factors; in the diagnostic perspective, clinicians should aim to confirm if the skin lesions are associated with COVID-19.

## 1. Introduction

In patients with COVID-19, previous skin lesions may be aggravated, and allergic reactions to the treatments used are possible [1]. Recently, publications of skin lesions that could correspond to the SARS-CoV-2 infection are increasing. Based on Galvan et al. [2], cutaneous lesions found in COVID-19 patients can be classified into five morphological patterns: maculopapular lesions (47%), pseudochilblains (19%), urticarial lesions (19%), vesicular eruptions (9%), and livedo or necrosis (6%). Other authors, such as Gottlieb et al. [3], performed an initial search of 1553 articles published, from which they selected a total of 41 articles for review. In addition to the dermatological manifestations described above, they added those of petechiae/purpura, and separated livedo racemosa from the distal ischaemia/necrosis category, placing them as two different sections. According to a recent systematic review by Conforti et al. [4] other lesion types were also reported, e.g., resembling erythema multiforme/generalized pustular figurate erythema/Stevens–Johnson syndrome or periocular types. Stefaniak et al. [5] has recently reviewed the possible etiology of itch in the context of COVID-19, as this common symptom may be associated not only with cutaneous manifestations of SARS-CoV-2 infection, but also stem from extensive use of chemicals (mainly disinfectants), personal protective equipment or arise due to psychosocial stress. Another peculiar subjective manifestation of COVID-19 is cutaneous hyperesthesia, as originally described by Krajewski et al. [6]. Suchonwanit et al. [7] have reported that COVID-19 onset is associated with skin manifestations, and most authors agree that the least affected topography is the face. Currently, there is still a lack of data supporting the relationship between dermatological manifestations and severity of the respiratory condition.

Clinicians must familiarize themselves with cutaneous manifestations of COVID-19 as the latter may occasionally pose as solitary signs of the disease. A better understanding of these may enable early diagnosis of COVID-19 or help guide prognosis and treatment. This manuscript provides our experience regarding skin lesions in patients with SARS-COV-2 infection during the COVID-19 pandemic.

## 2. Material and Methods

We performed a retrospective study, wherein all patients were studied, examined, and diagnosed by dermatologists from the different participating countries. Thirty-one patients from five countries (Argentina, Dominican Republic, India, Mexico, and Spain) diagnosed of COVID-19 with multiple skin lesions were enrolled in the present study. Exclusion criteria encompassed the lack of patients’ or their caregivers consent to participate in the study, as well as of the incompleteness of the clinical data obtained. The study was executed based on the CARE guidelines on case reports.

Taking into account that patient data were collected during the health emergency period with low sensitivity of some diagnostic tests, or the data were scarce, we proposed to include cases with a clinical diagnosis of the disease. All statistical analyses were performed using the SPSS software (ver22, IBM Corp., Armonk, NY, USA). Data were analyzed using the chi-squared test, Student’s *t*-test, and one-way analysis of variance, *p*-value for statistical significance 95%. Binary logistic regression was used to calculate the odds ratio (OR).

This study was approved by the Institutional Review Board of the Dr. Manuel Gea González General Hospital and the ethical committees of the different participating countries. Written informed consent for each procedure and for publication was obtained from all patients and their families. 

## 3. Results

**Figure 1:**
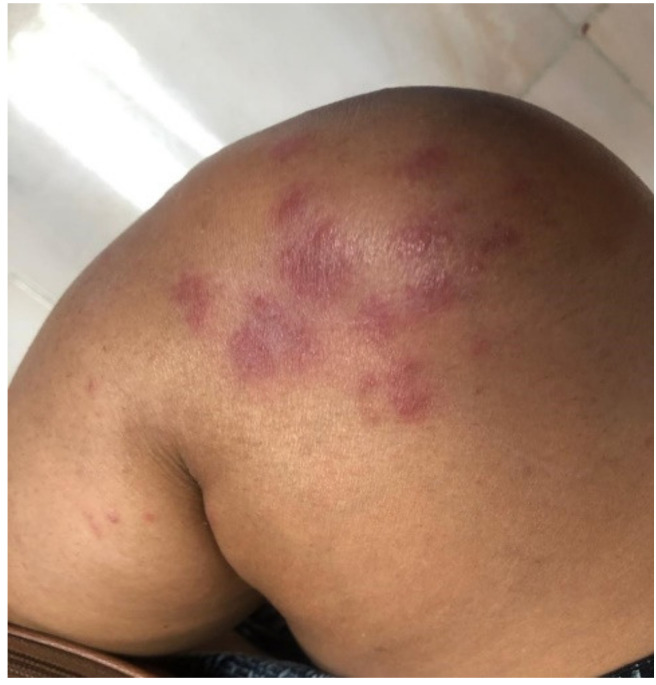
Clinical features of a 16-week pregnant patient diagnosed of COVID-19 with dusky purpuric patches on the knee.

We collected data from 33 patients from May 2020. Two patients were excluded due to missing or incomplete clinical information. Consequently, our series consisted of 31 COVID-19 patients from five different countries. The sociodemographic and clinical characteristics are shown in Table 1. The mean age of our patients was 45.55 ± 20.18 years, with a range of 3 months to 81 years. The majority of our patients were males (63.60%). Following the morphological patterns already described by previous authors, we classified the lesions into 7 different sections: maculopapular rashes observed in five (16.10%) patients; urticarial manifestations in 8 (25.80%); pseudochilblains (22.60%); distal petechiae/purpura (6.50%); ischemia and necrosis (6.50%); livedo racemosa (12.90%); and others (9.70%) which included patients whose lesions were aggravated by the COVID-19 treatment. Considering the occupation of our patients, the most affected were manual laborers (29.1%), followed by businessmen/women (22.60%) and students (16.10%). In terms of dermatological manifestations, the most frequently observed in our series were urticarial type lesions presenting as erythema and hives, which were clinically indistinguishable from acute urticaria. We emphasize that the lesions were fixed throughout the process. The second most frequent manifestation were pseudochilblains in the form of erythematous lesions, clinically similar to perniosis and initially asymptomatic, although some patients subsequently experienced pain. No patient had a history of rheumatic disease, lupus erythematosus, Raynaud’s phenomenon, acrocyanosis, or chilblains. We found no relationship between the manifestations and the concomitant treatments the patients received or the climatology of the countries of residence. The lesions were of a few millimeters and well defined, with the most frequent locations on the hands and feet, typically the lateral and posterior surfaces, the fingertips, and on the soles and heels. They were also observed more frequently in young, pauci-symptomatic patients, and occurred later in the disease course, usually several days after the onset of general symptoms. One of the patients was 16 weeks pregnant, and as far as we have followed, the pregnancy continued with its normal course (Figure 1).

**Figure 2:**
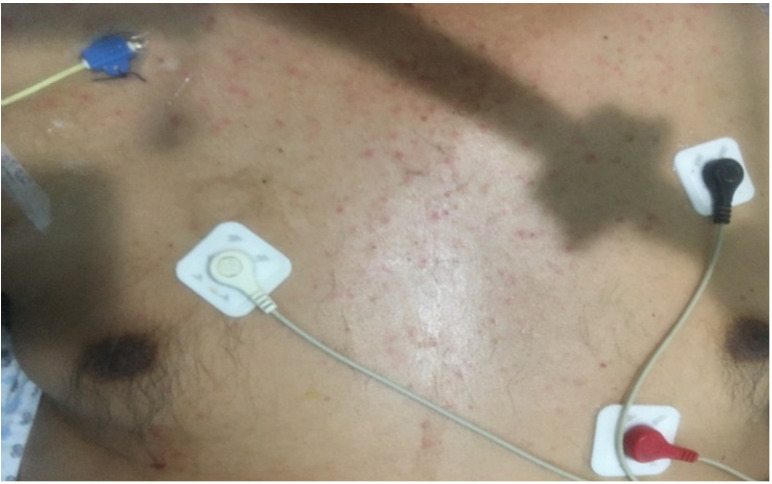
Erythematous, confluent, and predominantly perifollicular maculopapular rash in a patient diagnosed with COVID-19 and admitted to critical care.

The third most common manifestation were maculopapular-type lesions, usually generalized, with characteristics similar to other viral exanthemas, found predominantly on the trunk, and possibly accompanied by itching. Such lesions usually started days after the respiratory symptoms, and progressed favorably without specific treatment in most cases (Figure 2). 

**Figure 3:**
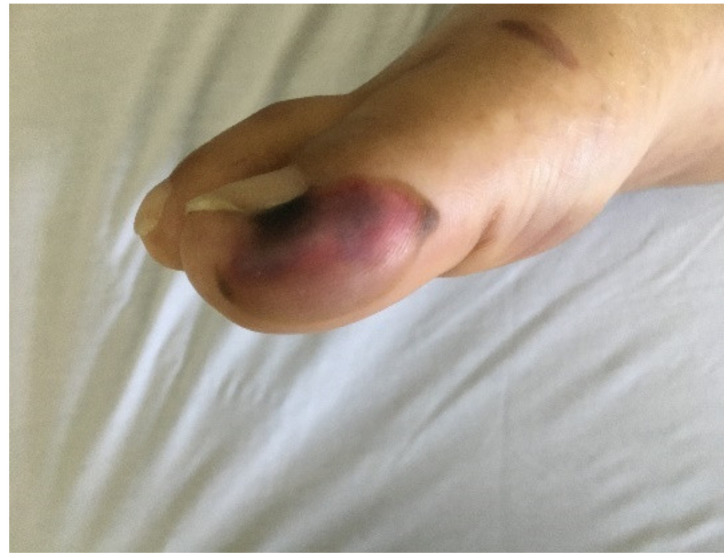
Skin lesions with a tendency to blister with subsequent necrosis on the right first toe of a patient confirmed for COVID-19.

Less common manifestations included livedo racemosa which was usually located unilaterally on the extremities. None of the patients presenting morbilliform rash had a history of taking medications in the days prior to the appearance of the rash, so they did not meet the criteria for morbiliform exanthem from drug eruption. Finally, petechiae/purpura and distal ischemia and necrosis (Figure 3) (6.50% each) were mostly located on the limbs and trunk. They were both associated with advanced stages of the disease.

The “others” section included two patients who presented with lesions that were aggravated as the disease progressed. One patient presented with a significant outbreak in terms of disease severity of lichen planus on the extremities and oral mucosa. The other patient, who had a history of herpes simplex virus (HSV) infection, suddenly developed ulcers and areas of necrosis in the mouth and genital areas five days after testing positive for COVID-19. Serological tests in the second patient revealed positive results for HSV-1 and HSV-2. The third patient included in the “others” section presented with telogen effluvium and was treated with hydroxychloroquine. The cutaneous manifestations in this last patient may have been related to their COVID-19 treatment.

**Figure 4:**
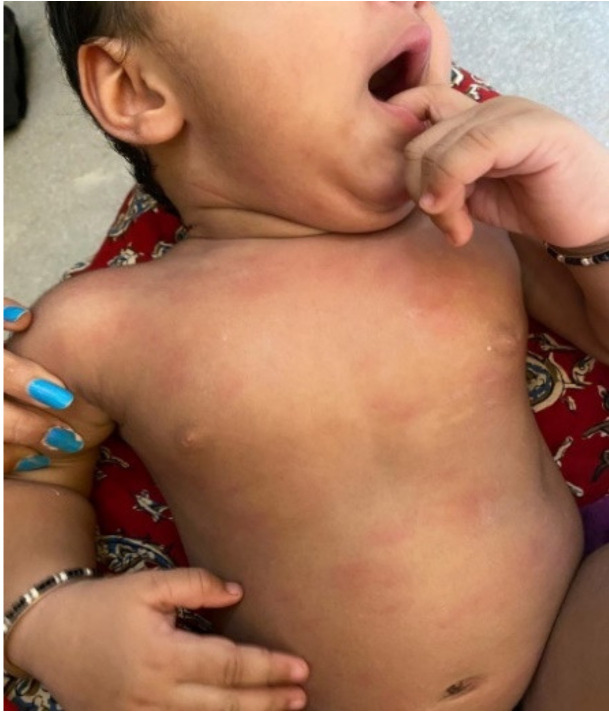
A one-year-old male diagnosed with COVID-19 with urticarial rashes initially affecting the face and upper extremities, followed by spreading to the trunk and lower extremities.

Our cohort also included pediatric patients with COVID-19. A one-year-old boy presented with urticarial lesions with mucosal involvement. His parents also had COVID-19. Blood tests did not reveal elevations in inflammatory parameters or significant impact on the general state; therefore, multisystem inflammatory syndrome in children (MIS-C) was excluded. The patient recovered satisfactorily with symptomatic treatment (Figure 4).

**Figure 5:**
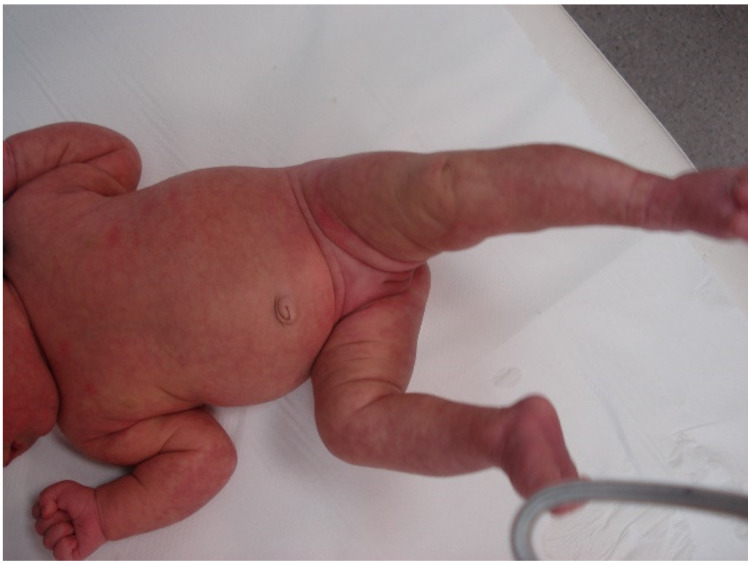
Generalized morbilliform rash and livedo racemosa both present at birth (the patient was diagnosed with pediatric multisystem inflammatory syndrome).

The second pediatric patient was a 3-month-old girl (Figure 5) who presented with a generalized morbilliform rash at birth, with affected palms and soles but sparing of the mucous membranes of the oral and nasal cavities. 

This rash settled in the area of livedo racemosa, which currently persists with more striking purple patchy areas on the trunk. The patient had respiratory failure and low-grade fever at birth. She was admitted to the intensive care pediatric unit of her referral hospital where she underwent cardiorespiratory monitoring, non-invasive ventilation, and enteral nutrition. Due to the presence of an increase in inflammation biomarkers, and subsequently the determination of positive IgG serology, MIS-C was diagnosed.

As SARS-CoV-2 detection by qRT-PCR on nasopharyngeal swabs was not performed in mild COVID-19 cases, skin manifestations of only 26 patients were utilized. Serological testing was performed in two patients, while two others were considered suspicious for SARS-CoV-2 infection. Thus, confirmatory laboratory diagnosis by means of the qRT-PCR technique was used in most of the participating countries, which represented 86.70% of the total study cohort.

The spectrum of cutaneous clinical manifestations of COVID-19 ranges from pseudochilblains in the less severe cases, to livedo racemosa, and finally to distal ischemia and necrosis presented in the most severe cases. Such patients require admission to the intensive care unit. In the patients included in our study, maculopapular eruptions commonly appeared in the early stages of COVID-19, while distal ischemia and necrosis patterns usually appeared in the late stages.

Treatment approaches differed between the countries. As these patients were studied at the beginning of the pandemic which was the period of health emergency, the most used drugs were paracetamol, non-steroidal anti-inflammatory drugs, azithromycin, hydroxychloroquine, and heparin. Subsequently, systemic corticosteroids with 6 mg dexamethasone every 24 h for 10 days were started; later, lopinavir, and ritonavir were added to the regime. None of our patients received tocilizumab or serum monoclonal antibodies (Ab) derived from patients who recovered from COVID-19. Sporadic cases were prescribed with antihistamines. The lack of survival of our patients was associated with pneumonia (95% CI: 2.986–286.425, sig 0.004, OR: 28.80), invasive mechanical ventilation use (95% CI: 6.331–1010.961, sig 0.001, OR: 80.00), and heparin use (95% CI: 3.701–395.304, sig 0.002, OR: 38.25). All three variables were, hence, considered risk factors that affected the probability of survival. 

## 4. Discussion

Cutaneous manifestations of COVID-19 have been poorly described in a limited number of case reports and case series. The first report of COVID-19-related cutaneous manifestations found in the literature is that of Recalcati et al. [8], who reported that 18 of their 88 COVID-positive patients presented with skin lesions. Estebanez et al. [9] reported the case of a 48-year-old woman (with confirmed COVID-19 under home isolation), who developed confluent erythematous and papular lesions on her heels that turned into indurated and itchy plaques, 13 days after the diagnostic test and ten days after the last intake of paracetamol. She denied any application of friction or pressure to the area. Other cases have described the association of livedo reticularis with microthrombosis and acrocyanosis [10]. One of the most frequent manifestations has been urticarial eruptions as reported by Bandhala Rajan et al. [11]. Hoenig et al. [12] described a patient with malar rash-erythema. In our opinion, with multiple possibilities of cutaneous manifestations, this new disease represents a “great simulator” as syphilis was back in its days. 

As for pregnant patients, not many cases of newborns of mothers who have suffered from COVID-19 have been reported in the literature. The risk of COVID-19 in newborns due to maternal transmission is undefined but appears to be low. In a work contributed by Ma et al. [13], all six reported cases of infants born to SARS-CoV-2-infected women achieved full recovery without requiring intubation. In another article published by Lei et al. [14], it involved nine pregnant women who delivered by caesarean section, and three who delivered vaginally. The pregnant patient in our series, to date, has not given birth, but the pregnancy is continuing its normal course with normal test and ultrasound scan results.

Concerning paediatric population, several cases of children and young people with acral lesions of the pseudochilblains type have been reported. They were mainly found on the toes, resolved spontaneously, and sometimes were not accompanied by other symptoms [15]. Many of these children, according to Andina et al. [16], had a history of close contact with positive COVID-19 relatives, while others were in contact with subjects with respiratory symptoms. It was, however, unusual for them to have been tested for this virus. Our first pediatric patient presented with urticarial-like lesions, and his parents were diagnosed with COVID-19 by PCR, but with the absence of skin lesions. The second child presented a moderate symptomatology compatible with MIS-C, in accordance with the clinical manifestations described in the current literature [17]. Regarding the concomitance with livedo racemosa, this can be explained by the fact that endothelial cells play an important role in patients with livedo racemosa, which has been similarly described in adult patients with COVID-19 [18]. Thus, according to Teuwen et al. [19], there is an important link between endothelial cells, viral infection, and inflammatory changes. It is unknown whether our observation concerned an atypical form of livedo reticularis or a different entity prior to infection of a congenital livedo racemosa by SARS-CoV-2. In a study by Roca-Ginés et al. [20] on a series of 20 patients from the La Fe hospital in Valencia, although COVID-19 infections could not be verified using laboratory techniques, the children presented with lesions similar to those described as pseudochilblains without any known medical cause in the midst of the pandemic. One possibility provided by the authors was that acrocyanosis lesions and perniosis were a subacute manifestation of the infection. Regarding the patient’s occupation, manual workers were mostly affected. This can be explained by the lack of protection and information in the most unprotected social strata, and in this we agree with Hawkins et al. [21]. Curiously, healthcare workers showed much smaller frequencies than those reported by other authors as Eyre et al. [22], although as we previously said, the sample is not large enough to draw conclusions in this regard. In our opinion, cutaneous manifestations of COVID-19 can be significantly polymorphic, occasionally non-specific, and ultimately resolve spontaneously. It seems clear that manifestations can appear due to two different mechanisms; lesions that present similar characteristics to viral exanthemas could be the consequence of an immune response to viral nucleotides, while the other large group stems from the systemic sequelae of COVID-19, especially vasculitis and thrombotic vasculopathy, as previously described [11]. 

In patients with COVID-19, worsening of previous skin lesions may also occur [23]. In our series, this happened in 2 patients, as demonstrated in Table 1. Treatment of these skin manifestations is usually symptomatic. Topical antihistamines, emollients, antiseptics, and corticosteroids may be prescribed in the case of rashes, urticarial eruptions, or vesicular eruptions. However, the policies and guidelines for COVID-19 treatment differ between countries, and some researchers have decided against administering treatment for asymptomatic patients and for those with mild symptoms, based on the premise that symptoms can disappear spontaneously after several days [24,25]. Patients with moderate to severe symptoms may receive multiple treatments, including systemic antibiotics/antivirals or interferons which are indicated in the appearance of generalized skin eruptions, and care should be taken when using these drugs as they further increase the complexity of the disease [26,27]. 

In conclusion, our study contributes new patient data to the existing literature. Using clinical data of patients from five different countries, the clinical characteristics were shown to corroborate with those observed in previous studies. Furthermore, there exist issues yet to be addressed, especially those related to the pathophysiological mechanisms underlying SARS-CoV-2 infection. Addressing these questions would lead to enhanced clarity about the nature of T-cell responses to SARS-CoV-2 infection. Careful definition of the different kinds of T-cell responses in COVID-19 and elucidation of risk factors, such as pre-existing conditions, comorbidities, race, and immune health, among others, is warranted. Moreover, little is known about whether SARS-CoV-2 favors the development of other viral processes and if it is competitive with other viruses having similar or different characteristics. Therefore, we believe it is necessary to investigate the appropriate treatment options for specific viral morphological patterns of presentation. Lastly, holistic knowledge of the spectrum of the skin lesions associated with COVID-19 is important to understand the virus spread.

## Figures and Tables

**Table 1:** Sociodemographic and clinical characteristics of skin lesions in COVID-19 patients.

	DERMATOLOGIC MANIFESTATIONS						
	Maculopapular Rash	Urticarial	Pseudochilblains	Petechiae/ Purpura	Distal Ischemia and Necrosis	Livedo Racemosa	Others
**COUNTRY**							
Argentina	1 (3.13)	0 (0)	0 (0)	0 (0)	0 (0)	0 (0)	1 (3.13)
Dominican Republic	1 (3.13)	4 (12.50)	0 (0)	0 (0)	0 (0)	0 (0)	0 (0)
India	0 (0)	2 (6.25)	0 (0)	0 (0)	0 (0)	0 (0)	0 (0)
Mexico	0 (0)	1 (3.13)	4 (12.50)	2 (6.25)	2 (6.25)	4 (12.50)	0 (0)
Spain	3 (9.38)	1 (3.13)	3 (9.38)	0 (0)	0 (0)	0 (0)	3 (9.38)
SEX							
Female	3 (9.38)	3 (9.38)	2 (6.25)	0 (0)	0 (0)	3 (9.38)	2 (6.25)
Male	2 (6.25)	5 (15.63)	5 (15.63)	2 (6.25)	2 (6.25)	2 (6.25)	1 (3.13)
**CIVIL STATE**							
Married	4 (12.50)	8 (25)	2 (6.25)	1 (3.13)	2 (6.25)	2 (6.25)	2 (6.25)
Single	1 (3.13)	0 (0)	5 (15.63)	0 (0)	0 (0)	2 (6.25)	1 (3.13)
Widowhood	0 (0)	0 (0)	0 (0)	1 (3.13)	0 (0)	1 (3.13)	0 (0)
**AGE (years)**							
0	1 (3.13)	0 (0)	0 (0)	0 (0)	0 (0)	1 (3.13)	0 (0)
1	0 (0)	1 (3.13)	0 (0)	0 (0)	0 (0)	0 (0)	0 (0)
15	0 (0)	0 (0)	1 (3.13)	0 (0)	0 (0)	0 (0)	0 (0)
17	0 (0)	0 (0)	1 (3.13)	0 (0)	0 (0)	0 (0)	0 (0)
25	0 (0)	1 (3.13)	0 (0)	0 (0)	0 (0)	0 (0)	0 (0)
28	1 (3.13)	0 (0)	0 (0)	0 (0)	0 (0)	0 (0)	0 (0)
29	0 (0)	1 (3.13)	0 (0)	0 (0)	0 (0)	0 (0)	0 (0)
30	0 (0)	0 (0)	0 (0)	0 (0)	0 (0)	0 (0)	1 (3.13)
31	0 (0)	1 (3.13)	0 (0)	0 (0)	0 (0)	0 (0)	0 (0)
32	0 (0)	1 (3.13)	0 (0)	0 (0)	0 (0)	0 (0)	0 (0)
36	1 (3.13)	0 (0)	0 (0)	0 (0)	0 (0)	0 (0)	0 (0)
39	0 (0)	0 (0)	1 (3.13)	0 (0)	0 (0)	0 (0)	0 (0)
43	0 (0)	0 (0)	0 (0)	0 (0)	0 (0)	0 (0)	1 (3.13)
47	0 (0)	0 (0)	1 (3.13)	0 (0)	0 (0)	0 (0)	0 (0)
49	0 (0)	2 (6.25)	0 (0)	0 (0)	0 (0)	1 (3.13)	0 (0)
51	0 (0)	0 (0)	0 (0)	0 (0)	1 (3.13)	0 (0)	0 (0)
52	1 (3.13)	1 (3.13)	1 (3.13)	0 (0)	0 (0)	0 (0)	0 (0)
55	0 (0)	0 (0)	1 (3.13)	0 (0)	0 (0)	0 (0)	0 (0)
60	0 (0)	0 (0)	0 (0)	1 (3.13)	0 (0)	0 (0)	0 (0)
61	0 (0)	0 (0)	0 (0)	1 (3.13)	0 (0)	0 (0)	0 (0)
62	0 (0)	0 (0)	1 (3.13)	0 (0)	1 (3.13)	0 (0)	1 (3.13)
63	1 (3.13)	0 (0)	0 (0)	0 (0)	0 (0)	0 (0)	0 (0)
67	0 (0)	0 (0)	0 (0)	0 (0)	0 (0)	1 (3.13)	0 (0)
77	0 (0)	0 (0)	0 (0)	0 (0)	0 (0)	1 (3.13)	0 (0)
81	0 (0)	0 (0)	0 (0)	0 (0)	0 (0)	1 (3.13)	0 (0)
**ACADEMIC LEVEL**							
Primary School	0 (0)	5 (16.67)	4 (13.33)	2 (6.67)	2 (6.67)	3 (10)	1 (3.33)
Secondary School	1 (3.33)	0 (0)	2 (6.67)	0 (0)	0 (0)	1 (3.33)	0 (0)
University Degree	3 (10)	3 (10)	1 (3.33)	0 (0)	0 (0)	0 (0)	2 (6.67)
**OCCUPATION**							
Driver	0 (0)	0 (0)	1 (3.33)	0 (0)	0 (0)	0 (0)	1 (3.33)
Handworker	0 (0)	3 (10)	1 (3.33)	1 (3.33)	2 (6.67)	2 (6.67)	0 (0)
Healthcare worker	0 (0)	1 (3.33)	0 (0)	0 (0)	0 (0)	0 (0)	2 (6.67)
Industrial	3 (10)	0 (0)	1 (3.33)	1 (3.33)	0 (0)	2 (6.67)	0 (0)
Professor	1 (3.33)	1 (3.33)	1 (3.33)	0 (0)	0 (0)	0 (0)	0 (0)
Student	0 (0)	2 (6.67)	2 (6.67)	0 (0)	0 (0)	0 (0)	0 (0)
Unemployed	0 (0)	1 (3.33)	1 (3.33)	0 (0)	0 (0)	0 (0)	0 (0)
**COVID-19 STATUS**							
Confirmed RT-PCR	2 (6.67)	8 (26.67)	6 (20)	2 (6.67)	2 (6.67)	4 (13.33)	2 (6.67)
Confirmed serological test	1 (3.33)	0 (0)	0 (0)	0 (0)	0 (0)	0 (0)	1 (3.33)
Suspected	1 (3.33)	0 (0)	1 (3.33)	0 (0)	0 (0)	0 (0)	0 (0)
**COUGH**	3 (13.04)	5 (21.74)	4 (17.39)	2 (8.70)	2 (8.70)	4 (17.39)	3 (13.04)
**DYSPNEA**	4 (16.67)	6 (25)	4 (16.67)	2 (8.33)	2 (8.33)	4 (16.67)	2 (8.33)
**FEVER**	5 (17.24)	7 (24.14)	6 (20.69)	2 (6.90)	2 (6.90)	4 (13.79)	3 (10.34)
**HEADACHE**	3 (11.54)	7 (26.92)	6 (23.08)	1 (3.85)	2 (7.69)	4 (15.38)	3 (11.54)
**ASTHENIA**	4 (15.38)	6 (23.08)	5 (19.23)	2 (7.69)	2 (7.69)	4 (15.38)	3 (11.54)
**NAUSEA, VOMITING, DIARRHOEA**	2 (50)	0 (0)	0 (0)	0 (0)	0 (0)	0 (0)	2 (50)
**ANOSMIA/AGEUSIA**	1 (25)	1 (25)	0 (0)	0 (0)	0 (0)	1 (25)	1 (25)
**PNEUMONIA**	1 (7.69)	1 (7.69)	3 (23.08)	1 (7.69)	2 (15.38)	4 (30.77)	1 (7.69)
**SMOKING**	3 (30)	3 (30)	1 (10)	0 (0)	1 (10)	1 (10)	1 (10)
**COPD**	0 (0)	0 (0)	0 (0)	0 (0)	0 (0)	1 (100)	0 (0)
**TYPE 2 DIABETES**	1 (11.11)	1 (11.11)	3 (33.33)	1 (11.11)	2 (22.22)	1 (11.11)	0 (0)
**ARTERIAL HYPERTENSION**	1 (16.67)	0 (0)	1 (16.67)	1 (16.67)	1 (16.67)	2 (33.33)	0 (0)
**PRESENCE OF CUTANEOUS SYMPTOMS**							
Pain	1 (2.13)	1 (2.13)	4 (8.51)	2 (4.26)	2 (4.26)	2 (4.26)	1 (2.13)
Burning	2 (4.26)	7 (14.89)	6 (12.77)	1 (2.13)	1 (2.13)	0 (0)	2 (4.26)
Itch	4 (8.51)	7 (14.89)	3 (6.38)	1 (2.13)	0 (0)	0 (0)	0 (0)
**DURATION OF CUTANEOUS LESIONS (days)**							
7	0 (0)	2 (6.25)	0 (0)	0 (0)	0 (0)	0 (0)	0 (0)
8	0 (0)	2 (6.25)	0 (0)	0 (0)	0 (0)	0 (0)	0 (0)
9	2 (6.25)	3 (9.38)	0 (0)	0 (0)	0 (0)	0 (0)	0 (0)
10	2 (6.25)	1 (3.13)	0 (0)	0 (0)	0 (0)	1 (3.13)	0 (0)
11	1 (3.13)	0 (0)	0 (0)	0 (0)	0 (0)	2 (6.25)	0 (0)
12	0 (0)	0 (0)	3 (9.38)	2 (6.25)	0 (0)	2 (6.25)	0 (0)
13	0 (0)	0 (0)	2 (6.25)	0 (0)	1 (3.13)	0 (0)	0 (0)
14	0 (0)	0 (0)	2 (6.25)	0 (0)	1 (3.13)	0 (0)	1 (3.13)
15	0 (0)	0 (0)	0 (0)	0 (0)	0 (0)	0 (0)	1 (3.13)
20	0 (0)	0 (0)	0 (0)	0 (0)	0 (0)	0 (0)	1 (3.13)
**TREATMENTS**							
Paracetamol/ Acetominophen	5 (6.02)	5 (6.02)	5 (6.02)	2 (2.41)	2 (2.41)	6 (14.53)	3(3.61)
Systemic corticosteroids	1 (1.20)	0 (0)	0 (0)	0 (0)	0 (0)	2 (2.41)	1 (1.20)
Antihistamines	3 (3.61)	2 (2.41)	0 (0)	0 (0)	0 (0)	1 (1.20)	1 (1.20)
Azithromycin	2 (2.41)	1 (1.20)	2 (2.41)	1 (1.20)	1 (1.20)	4 (4.82)	1 (1.20)
NSAIDs	0 (0)	0 (0)	4 (4.82)	1 (1.20)	1 (1.20)	3 (3.61)	0 (0)
Hydroxychloroquine	1 (1.20)	0 (0)	2 (2.41)	2 (2.41)	1 (1.20)	2 (2.41)	1 (1.20)
Lopinavir, ritonavir	0 (0)	0 (0)	0 (0)	0 (0)	0 (0)	0 (0)	1 (1.20)
Heparin	0 (0)	1 (1.20)	3 (3.61)	2 (2.41)	2 (2.41)	4 (4.82)	1 (1.20)
**INVASIVE MECHANICAL VENTILATION (injuries)**	1 (9.09)	0 (0)	3 (27.27)	1 (9.09)	2 (18.18)	4 (36.36)	0 (0)
**NOREPINEPHRINE (detection of lesions while using amines)**	0 (0)	0 (0)	1 (20)	1 (20)	1 (20)	2 (40)	0 (0)
**PATIENT SURVIVAL**	4 (20)	7 (35)	4 (20)	1 (5)	1 (5)	0 (0)	3 (15)

Number of patients (%); COPD: Chronic obstructive pulmonary disease; NSAIDs: Nonsteroidal anti-inflammatory drugs.

## Data Availability

The data presented in this study are available on reasonable request from the corresponding author.

## References

[1] Yao X.H., Li T.Y., He Z.C., Ping Y.F., Liu H.W., Yu S.C., Mou H.M., Wang L.H., Zhang H.R., Fu W.J. (2020). A pathological report of three COVID-19 cases by minimal invasive autopsies. Zhonghua Bing Li Xue Za Zhi.

[2] Galván Casas C., Català A., Carretero Hernández G., Rodríguez-Jiménez P., Fernández-Nieto D., Rodríguez-Villa Lario A., Navarro Fernández I., Ruiz-Villaverde R., Falkenhain-López D., Llamas Velasco M. (2020). Classification of the cutaneous manifestations of COVID-19: A rapid prospective nationwide consensus study in Spain with 375 cases. Br. J. Dermatol..

[3] Gottlieb M., Long B. (2020). Dermatologic manifestations and complications of COVID-19. Am. J. Emerg. Med..

[4] Conforti C., Dianzani C., Agozzino M., Giuffrida R., Marangi G.F., Meo N.D., Morariu S.H., Persichetti P., Segreto F., Zalaudek I. (2020). Cutaneous manifestations in confirmed COVID-19 patients: A systematic review. Biology.

[5] Stefaniak A.A., Białynicki-Birula R., Krajewski P.K., Matusiak Ł., Goldust M., Szepietowski J.C. (2020). Itch in the era of COVID-19 pandemic: An unfolding scenario. Dermatol. Ther..

[6] Krajewski P.K., Szepietowski J.C., Maj J. (2020). Cutaneous hyperesthesia: A novel manifestation of COVID-19. Brain Behav. Immun..

[7] Suchonwanit P., Leerunyakul K., Kositkuljorn C. (2020). Cutaneous manifestations in COVID-19: Lessons learned from current evidence. J. Am. Acad. Dermatol..

[8] Recalcati S. (2020). Cutaneous manifestations in COVID-19: A first perspective. J. Eur. Acad. Dermatol. Venereol..

[9] Estébanez A., Pérez-Santiago L., Silva E., Guillen-Climent S., García-Vázquez A., Ramón M.D. (2020). Cutaneous manifestations in COVID-19: A new contribution. J. Eur. Acad. Dermatol. Venereol..

[10] Mirza F.N., Malik A.A., Omer S.B., Sethi A. (2020). Dermatologic manifestations of COVID-19: A comprehensive systematic review. Int. J. Dermatol..

[11] Bandhala Rajan M., Kumar M.P., Bhardwaj A. (2020). The trend of cutaneous lesions during COVID-19 pandemic: Lessons from a meta-analysis and systematic review. Int. J. Dermatol..

[12] Hoenig L.J., Pereira F.A. (2020). Eruption as a clinical manifestation of COVID-19: Photographs of a patient. Clin. Dermatol..

[13] Ma X., Zhu J., Du L. (2020). Neonatal management during coronavirus disease (COVID-19) outbreak: Chinese experiences. NeoReviews.

[14] Lei D., Wang C., Li C., Zhou Z., Liu S., Rong Z. (2020). Clinical characteristics of COVID-19 in pregnancy: Analysis of nine cases. Chin. J. Perinat. Med..

[15] Colonna C., Monzani N.A., Rocchi A., Gianotti R., Boggio F., Gelmetti C. (2020). Chilblain-like lesions in children following suspected COVID-19 infection. Pediatr. Dermatol..

[16] Andina D., Noguera-Morel L., Bascuas-Arribas M., Gaitero-Tristán J., Alonso-Cadenas J.A., Escalada-Pellitero S., Hernández-Martín Á., de la Torre-Espi M., Colmenero I., Torrelo A. (2020). Chilblains in children in the setting of COVID-19 pandemic. Pediatr. Dermatol..

[17] Feldstein L.R., Rose E.B., Horwitz S.M., CDC COVID-19 Response Team (2020). Multisystem inflammatory syndrome in U.S. children and adolescents. N. Engl. J. Med..

[18] Sajjan V.V., Lunge S., Swamy M.B., Pandit A.M. (2015). Livedo reticularis: A review of the literature. Indian Dermatol. Online J..

[19] Teuwen L.A., Geldhof V., Pasut A., Carmeliet P. (2020). COVID-19: The vasculature unleashed. Nat. Rev. Immunol..

[20] Roca-Ginés J., Torres-Navarro I., Sánchez-Arráez J., Abril-Pérez C., Sabalza-Baztán O., Pardo-Granell S., Cózar V.M.I., Botella-Estrada R., Évole-Buselli M. (2020). Assessment of acute acral lesions in a case series of children and adolescents during the covid-19 pandemic. JAMA Dermatol..

[21] Hawkins D. (2020). Differential occupational risk for COVID-19 and other infection exposure according to race and ethnicity. Am. J. Ind. Med..

[22] Eyre D.W., Lumley S.F., O’Donnell D., Campbell M., Sims E., Lawson E., Warren F., James T., Cox S., Howarth A. (2020). Differential occupational risks to healthcare workers from SARS-CoV-2 observed during a prospective observational study. Elife.

[23] Young S., Fernandez A.P. (2020). Skin manifestations of COVID-19. Cleve Clin. J. Med..

[24] Henry D., Ackerman M., Sancelme E., Finon A., Esteve E. (2020). Urticarial eruption in COVID-19 infection. J. Eur. Acad. Dermatol. Venereol..

[25] Najarian D.J. (2020). Morbilliform exanthem associated with COVID-19. JAAD Case Rep..

[26] Sanghvi A.R. (2020). COVID-19: An overview for dermatologists. Int. J. Dermatol..

[27] Martinez-Lopez A., Cuenca-Barrales C., Montero-Vilchez T., Molina-Leyva A., Arias-Santiago S. (2020). Review of adverse cutaneous reactions of pharmacologic interventions for coronavirus disease 2019 (COVID-19): A guide for the dermatologist. J. Am. Acad Dermatol..

